# Apatinib-induced protective autophagy and apoptosis through the AKT–mTOR pathway in anaplastic thyroid cancer

**DOI:** 10.1038/s41419-018-1054-3

**Published:** 2018-10-09

**Authors:** Haoran Feng, Xi Cheng, Jie Kuang, Lingxie Chen, Stanley Yuen, Minmin Shi, Juyong Liang, Baiyong Shen, Zhijian Jin, Jiqi Yan, Weihua Qiu

**Affiliations:** 1 Department of General Surgery, Ruijin Hospital, Shanghai Jiao Tong University School of Medicine, 200025 Shanghai, China; 2 Shanghai Institute of Digestive Surgery, Ruijin Hospital, Shanghai Jiao Tong University School of Medicine, 200025 Shanghai, China; 3 Biology Chemistry Major, University at Albany, New York, NY USA

## Abstract

Apatinib, an inhibitor of vascular endothelial growth factor receptor-2, has been shown to promote anti-cancer action across a wide range of malignancies, including gastric, lung, and breast cancers. Our previous study showed that apatinib increases apoptosis in anaplastic thyroid carcinoma (ATC), but the direct functional mechanism of tumor lethality mediated by apatinib is still unknown. In this study, we demonstrated that apatinib induced both autophagy and apoptosis in human ATC cells through downregulation of p-AKT and p-mTOR signals via the AKT/mTOR pathway. Moreover, inhibition of apatinib-induced autophagy increased apatinib-induced apoptosis in ATC cells, and additional tumor suppression was critically produced by the combination of apatinib and the autophagy inhibitor chloroquine in vivo and in vitro. These findings showed that both autophagy and AKT/mTOR signals were engaged in ATC cell death evoked by apatinib. ATC patients might benefit from the new anti-cancer drug, and molecular targeted treatment in combination with autophagy inhibitors shows promise as a treatment improvement.

## Introduction

Thyroid cancer is one of the most common malignant endocrine tumors. More than 50,000 new thyroid cancer cases would be diagnosed in 2018, and the quantity of cases shows an obvious increasing trend^1^. The incidence of anaplastic thyroid carcinoma (ATC) is 1.5% in all thyroid cancers, and it is the major cause of all thyroid carcinoma-related deaths with a median survival time of 3–9 months due to high levels of extrathyroidal invasion, distant metastasis and resistance to conventional treatment^2–4^. In all, ATC patients require more effective therapy in addition to surgery and radioactive iodine therapy.

Apatinib, a small-molecule inhibitor of vascular endothelial growth factor receptor-2 (VEGFR-2), can induce apoptosis and suppress tumor proliferation in a variety of tumors^5–7^. This drug has shown promising results in gastric cancer patients who failed standard chemotherapy^8,9^. In addition, clinical trials that include breast cancer, esophageal cancer, colorectal cancer, liver cancer, and non-small cell lung cancer (NSCLC) are currently under investigation^10–14^.

Apoptosis and autophagy are the two main mechanisms that cause programmed cell death (PCD)^15^. Unlike apoptosis, autophagy is considered a double-edged sword that depends on the nature and cellular context of the stimuli^16,17^. The role of autophagy in cancer is complex. Under certain stress conditions, upregulation of autophagy might lead to cell death^18,19^. With the selective degradation of cellular components, autophagy also supplies a cell-survival pathway, maintaining the recycling of nutrients and the generation of energy in all eukaryotes^20–22^.

In our previous study, we proved that apatinib could be a potential therapeutic strategy for ATC in vivo and in vitro^23^. However, the detailed regulation mechanism still needs further illustration. In this study, we confirmed that apatinib could induce autophagy and apoptosis through the AKT/mTOR pathway in ATC cells and that autophagy inhibition by chloroquine (CQ) could significantly enhance the anti-cancer effects of apatinib. These findings offer sequential and valid complementary evidence to our initial apatinib research.

## Materials and methods

### Cell culture and compounds

Human ATC cell lines KHM-5M and C643 were purchased from the China Center for Type Culture Collection (CCATCC, China). The C643 and KHM-5M cells were cultured in RPMI-1640 medium supplemented with 10% foetal bovine serum (Gibco, USA) at 37 °C in 5% CO_2_ (Shanghai Medical Instruments, China). Apatinib was obtained from Hengrui Medicine Co. Ltd. (Jiangsu, China), dissolved in DMSO and diluted with 1640 medium to the desired concentration with a final DMSO concentration of 0.1% for in vitro studies. Prior to each treatment, cells were plated overnight and displayed a similar subconfluently density at the time of drug exposure. The SC79, CQ, and rapamycin were purchased from Sigma-Aldrich Chemical Company (St. Louis, MO, USA) and were dissolved in PBS and diluted with RPMI-1640 to the desired concentration. Bafilomycin A1 (Baf A1) was obtained from Selleck Chemicals (Houston, TX, USA).

### Establishment of stable cell lines for autophagy analyses

Lentiviral packaging was performed as previously described^24^. In brief, 24 h prior to transfection, C643 and KHM-5M cells in the logarithmic growth phase were trypsinized and adjusted to 1.0 × 10^6^ per ml. The cells were reseeded into 15-cm cell culture dishes and cultured for 24 h prior to transfection. The cells were 90–95% confluent on the day of transfection. Recombinant viral vectors encoding GFP-RFP- HLC3 (Jiman, China) were transfected into C643 and KHM-5M cells according to the manufacturer’s instructions. After 48 h, the expression of GFP and RFP was detected under an epifluorescence microscope (Olympics IX 71). Antibiotic-resistant colonies were selected on LB-puromycin agar plates for 2 weeks. After colony selection and further propagation, the stable cell line plasmid was maintained in RPMI-1640 (Sigma) at 37 °C.

### Cell viability assay and colony formation assay

The cytotoxicity of apatinib was estimated using the CCK-8 assay (Cell Counting Kit-8, Dojindo, Japan). Approximately 3000 cells were plated in 96-well plates and cultured in a 37 °C/5% CO_2_ incubator for 24 h before treatment. The cells were treated with apatinib at 0, 5, 10, 20, 40, and 80 µM for 24, 48, or 72 h, respectively. DMSO was added to the cultures as a solvent control. At the test point, 100 μl Cell Counting Kit-8 (CCK-8) solution was added into each well, and the plate was incubated at 37 °C for 2 h followed by OD detection using a spectrophotometer. For the colony formation assay, 1000 cells were plated in 6-well plates and cultured at 37 °C for 2 weeks. The colonies were visualized by staining with 0.1% crystal violet in methanol for 30 min. For the colony formation assay, 1000 cells suspended in 0.7% soft agar were plated on the surface of 1.2% soft agar in a 6-well plate and cultured at 37 °C for 2 weeks.

### Confocal microscopy

Cells were fixed with 4% paraformaldehyde (Sigma) for 20 min. Subsequently, the cells were permeabilized with 0.1% Triton X-100 (Sigma) for 15 min at room temperature, washed with PBS and blocked with PBS containing 0.5% (w/v) bovine serum albumin (BSA) and 0.15% (w/v) glycine (BSA buffer) for 1 h at room temperature. The slices were treated with DAPI (Sigma) and visualized by confocal microscopy (Zeiss, LSM510).

### Cell transfection with siRNA

ATG7 is essential for the autophagy conjugation system, autophagosome formation, and starvation-induced degradation of proteins and organelles in mammalian cells^25^. Therefore, C643 and KHM-5M cells were pre-treated with ATG7-siRNA interference to assess the contribution of autophagy. The ATG7-targeting sense sequence and the Universal Negative Control-siRNA were purchased from Invitrogen (12935-400, Invitrogen, Carlsbad, CA). The human ATG7 sequence (5′-GGAGATCACAGCATCTATCCTT-3′) was cloned into the *Bam*H1 and *Eco*R1 sites of the pGSU6-GFP vector (GTP600300, Genlantis, San Diego, CA). The siRNA plasmids were transfected using Lipofectamine 2000 (Invitrogen). Forty-eight hours after transfection, cells were collected via flow cytometry sorting. The irrelevant nucleotides did not target any annotated human genes and served as a negative control. The cells were cultured and treated by apatinib (20 μM) for an additional 24 h.

### Flow cytometry assay

Flow cytometric assays of apoptosis were performed as previously described^26^. In brief, the Annexin V-FITC Apoptosis Detection kit (BD Pharmingen, USA) was used to detect the apoptosis of cells. Cells were treated with 0, 10, 20, and 40 µM apatinib for 24 h. Both attached and the floating cells were harvested, washed twice with ice-cold PBS, and suspended in 100 µl binding buffer. The cells were stained with 3 µl Annexin V-FITC and 5 µl propidium iodide (PI) and incubated at room temperature for 15 min in the dark. Finally, 300 µl 1× binding buffer was added to each sample of cells. Apoptosis was analyzed by flow cytometry using the FACSCalibur system (BD Biosciences, USA).

### Electron microscopy

After treatment, C643 and KHM-5M cells were immediately fixed in 2% glutaraldehyde and 2% paraformaldehyde in 0.1 mol/l sodium phosphate buffer (pH 7.4) at 4 °C for 3 h. The samples were embedded in a mixture of epon 618 and epoxypropane after post-fixing in 1% osmium tetroxide in the same buffer for 2 h and gradual dehydration in alcohols. Semi-thin sections were stained with toluidine blue, and ultrathin sections were stained with 5% uranyl acetate and Reynold’s lead citrate. Sections were examined on a Hitachi electron microscope equipped with a digital camera. For morphometric analysis, at least three independent experiments were performed.

### Western blotting

After treatment, cells were harvested at various time intervals and digested in RIPA buffer in the presence of Protease Inhibitor (Pierce, USA) and Protein Phosphatase Inhibitor (New Cell & Molecular Biotech, China) Cocktail. Total protein was extracted and quantified using BCA Protein Assay Kit (Pierce, Rockford, USA). Proteins were separated by SDS-PAGE and transferred to a PVDF membrane (Tanon, China). Blots were probed with anti-PI3K (1:1000), anti-pPI3K (1:1000), anti-mTOR (1:1000), anti-p-mTOR (1:1000), anti-AKT (1:1000), anti-p-AKT (1:1000), anti-p62/SQSTM-1 (1:1000), anti-Beclin 1 (1:1000), anti-ATG7 (1:1000), anti-Bax (1:1000), anti-Bcl2 (1:1000), anti-PARP (1:1000), anti-Cleaved-caspase 3 (1:1000), anti-Caspase 3 (1:1000), anti-Cleaved-PARP (1:1000), anti-LC3 (1:1000) (all from Cell Signalling Technology, USA), and GAPDH (Abcam, 1:10,000) overnight at 4 °C. GAPDH was used as the internal control. Goat anti-rabbit or goat anti-mouse horseradish-peroxidase-conjugated IgG was used as the secondary antibody (Santa Cruz Biotechnology), and samples were incubated at room temperature for 1 h. Bands were observed using enhanced chemiluminescence (Pierce). Band density was measured by densitometry, quantified using the public domain NIH Image J software (open-source Image J software is available at http://rsb.info.nih.gov/ij/) and normalized to an indicated sample in the identical membrane.

### In vivo PC xenograft tumor model

Four-week-old male BALB/c nude mice were purchased from the Institute of Zoology, Chinese Academy of Sciences of Shanghai. All experiments were performed in accordance with the official recommendations of the Chinese Zoological Society, and animals received humane care according to the criteria outlined in the “Guide for the Care and Use of Laboratory Animals”. A suspension containing 1 × 10^6^ KHM-5M cells was subcutaneously injected into the right flanks of the nude mice. After ~2 weeks, when the tumors reached ~5 mm, all mice were randomly divided into four groups (4 in each group, group 1: Vehicle-only solution; group 2: CQ 60 mg/kg; group 3: Apatinib 50 mg/kg; and group 4: CQ 60 mg/kg + apatinib 50 mg/kg). These compounds, dissolved in NS (normal saline solution), were administered via daily oral gavage per day. The tumor dimensions were measured using a Vernier caliper, and the tumor volume was calculated using the following formula: *V* = *π*/6 × (*W*^2^ × *L*). Tumor size and body mass were recorded twice per week. Three days after the last injection, the animals were killed by cervical decapitation, and tumors were removed and weighed. Samples were prepared for immunoblot analysis and TUNEL staining.

### Immunohistochemistry and TUNEL assay

Immunohistochemistry analysis of xenograft tumors in nude mice was conducted as previous described^27^. In brief, tumor tissue sections with 4 μm thickness were cut from paraffin-embedded tissue blocks, deparaffinized and rehydrated, and treated with 0.01 mol/l citrate buffer (pH 6) for antigen retrieval. After inhibition of endogenous peroxidase activity for 30 min with methanol containing 0.3% H_2_O_2_, the sections were incubated with rabbit anti-caspase-3 (1:200, Santa Cruz), and anti-Ki-67 (1:200, Santa Cruz) at 4 ℃ overnight. After incubation with HRP-labeled secondary antibody, apoptosis was identified using with the In Situ Cell Death Detection Kit, Fluorescein (Roche Applied Science, USA). Nuclei were detected by DAPI staining.

### Statistics

Data are expressed as the means ± SD. Analysis of variance (ANOVA) and Student’s *t*-test were chosen for comparison among groups. The Mann–Whitney *U*-test was applied in comparison of tumor volume. Categorical data were evaluated with the *χ*^2^ test or Fisher’s exact test. *P*-values <0.05 were considered significant. Statistical analyses were processed using SPSS13.0 (SPSS Inc. Chicago, IL, USA).

## Results

### Apatinib inhibits viability of ATC cells

**Fig. 1:**
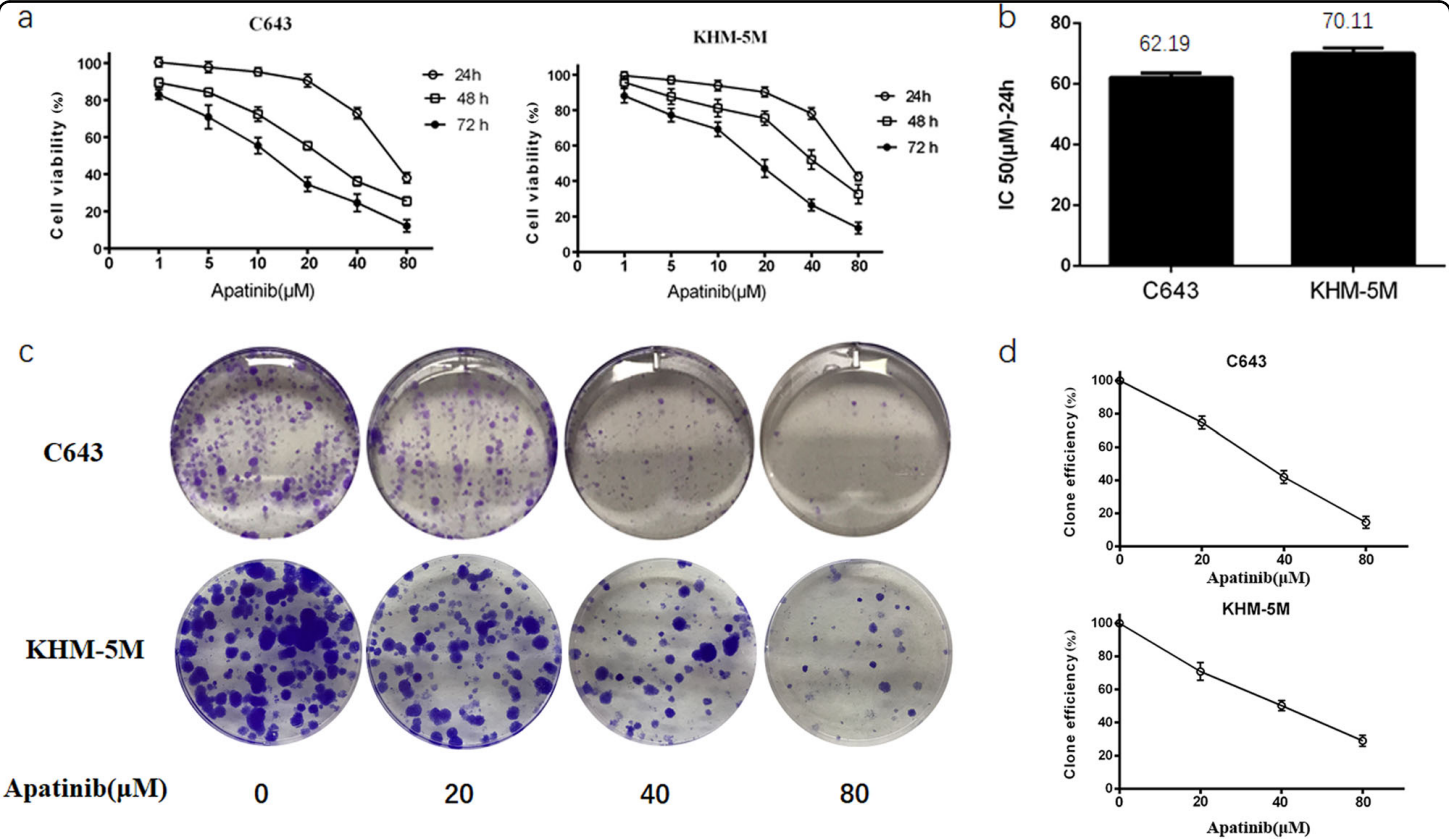
Apatinib inhibited the proliferation of ATC cells. **a** ATC cells were cultured in a series of concentration of apatinib for 24, 48, and 72 h. Cell viability was assessed by CCK-8 assay. **b** The IC-50 of apatinib treated in C643 and KHM-5M for 24 h. **c**, **d** Representative images of C643 cell contact-dependent clone formation. Each experiment is representative of three independent experiments

Our previous study showed that apatinib treatment could significantly inhibit the proliferation of ATC cell lines in vitro^23^. The IC-50 values for C643 and KHM-5M with apatinib were at the intermediate level of all ATC cell lines. Those data were established in our previous study, and the IC-50 values for C643 and KHM-5M were 62.68 and 70.83 for 24 h, respectively^23^. In this study, C643 and KHM-5M were incubated with a series of concentrations of apatinib for 24, 48, and 72 h. Apatinib concentrations ranging from 5 to 20 μM did not significantly affect cell viability, whereas cytotoxicity increased dramatically at dosages greater than 40 μM (Fig. 1a). Therefore, based on the CCK8 assay and to assess the role of apatinib-induced autophagy in ATC cells, the working concentration of apatinib was defined at 20 μM for 24 h without resulting in obvious cell necrosis. The contact-dependent growth assay by cloning formation was performed to further confirm the effect of apatinib on inhibiting the proliferation of ATC cells. We identified fewer clone numbers in C643 and KHM-5M cells after apatinib treatment (Fig. 1c, d). Additionally, the decreasing trend in clone numbers is parallel to the concentration of apatinib treatment. When the apatinib concentration was greater than 40 μM, the cytotoxicity increased dramatically (Fig. 1b–d). All of these results confirmed the efficiency of apatinib in ATC cell inhibition and the IC-50 value in vitro.

### Increasing the apatinib level increases autophagosome formation

**Fig. 2:**
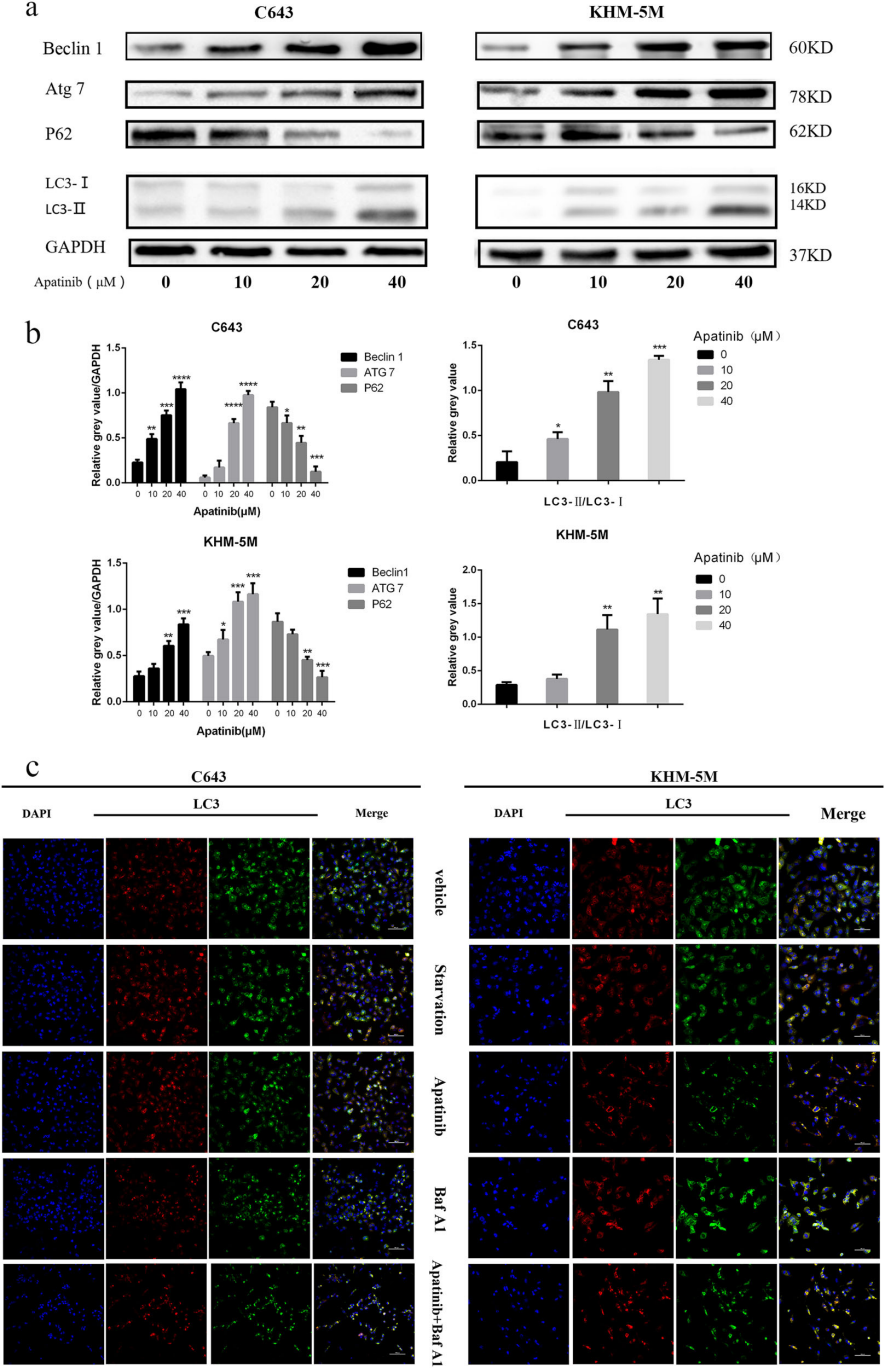

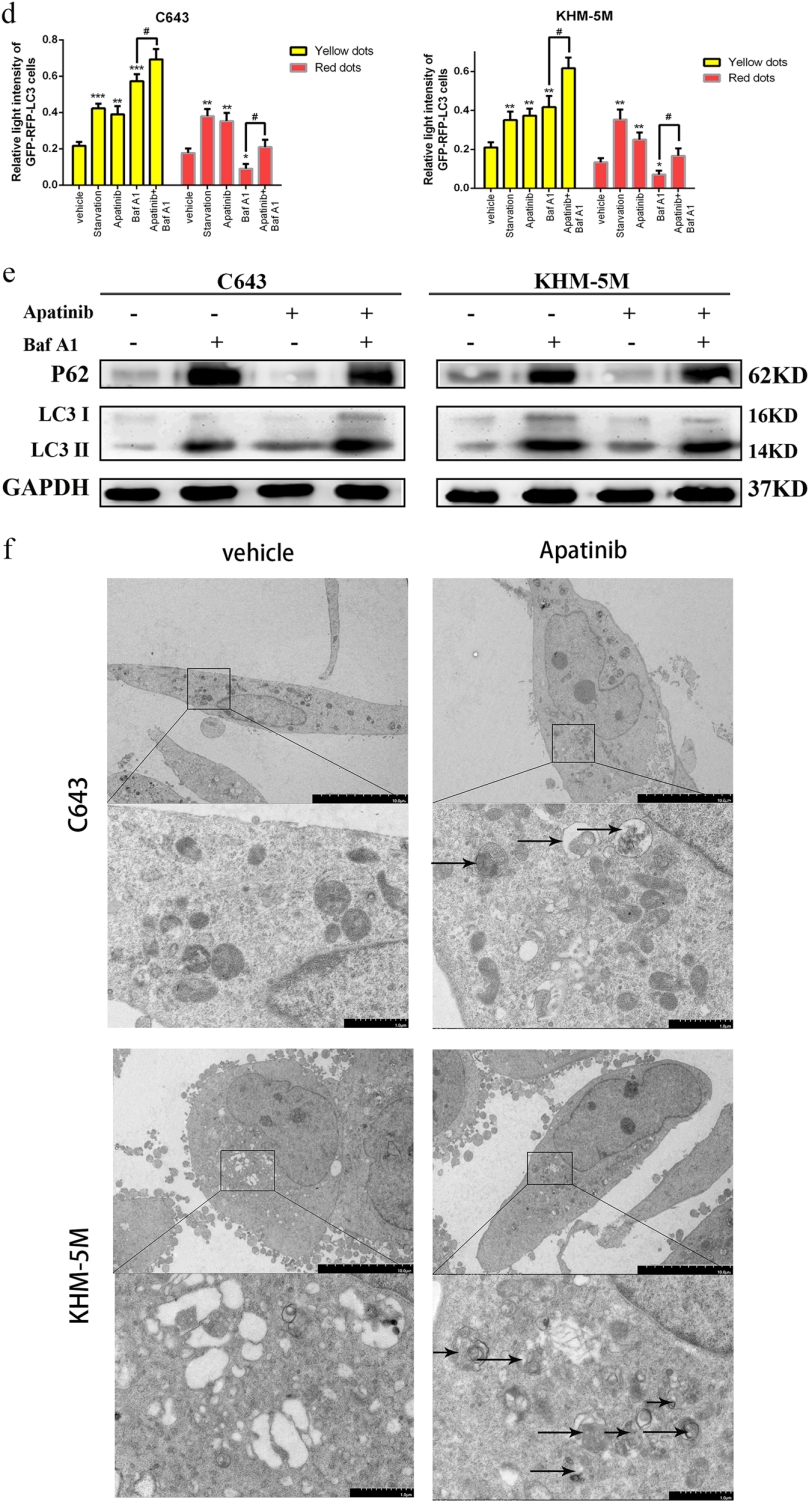
Apatinib induced autophagy in ATC cells. **a** C643 and KHM-5M cells were treated with a serious concentration of apatinib for 24 h. The Beclin 1, ATG7, P62, LC3-II, and GAPDH expressions were detected by western blot. **b** Densitometric analysis of the immunoblot reported in Fig. 2a. **c** Representative images of early-autophagosomes (yellow dots generated from overlapping GFP and RFP puncta) shown as yellow points and late autolysosomes (red dots generated from RFP puncta) shown as red points. C643 RFP-GFP-LC3 and KHM-5M RFP-GFP-LC3 cells (transfected with the RFP-GFP-hLC3 lentivirus) treated with 20 μM apatinib for 24 h, starvation or 50 nM Baf A1 for 12 h. **d** Densitometric analysis of the picture in Fig. 2c. **e** ATC cells were treated as such Fig. 2c, the LC3, P62, and GAPDH were detected by western blot. **f** Electron microscopy showed C643 and KHM-5M cells in the presence of apatinib (20 μM/24 h). *p < 0.05; **p < 0.01, ***p < 0.001, ****p < 0.0001 compared with vehicle group. ^#^*p* < 0.05; ^##^*p* < 0.01, ^###^*p* < 0.001, ^####^*p* < 0.0001. Arrows: Autophaosomes/autolysosomes

Three different independent methods were used to examine autophagosome formation. First, western blot analysis was used to detect the expression of Beclin 1, LC3, ATG7, and P62. LC3 lipidation is a specific marker of autophagosomes in mammalian cells, and it is widely used in autophagy measurement. P62 is a hallmark of autophagic flux, and its expression level is closely associated with autophagy flux trigger and inhibition. Our results showed that the expression of LC3-II (LC3 lipidation), Beclin 1 and ATG7 were parallel to the increase in apatinib concentration, whereas P62 was consequently decreased (Fig. 2a, b). To further detect autophagic flux, Baf A1 was administered in ATC with or without apatinib. Our results showed p62 and LC3-II were increased with Baf A1/apatinib treatment than apatinib solo (Fig. 2e). Based on these results, we confirmed that apatinib could induce autophagosome formation in ATC cells in a dose-dependent manner. In addition, GFP-RFP-hLC3 fluorescence was used to detect autophagic flux. The lentivirus-encoding GFP-RFP-hLC3 fluorescent gene was transfected into ATC cells. The GFP-RFP-hLC3 fusion protein was dispersed in the cytoplasm, showing yellow-green spots (overlay of green and red fluorescence) under fluorescence microscopy. In autolysosomes, RFP was stably expressed with GFP quenching, and therefore, autolysosomes displayed red spots in contrast to yellow-green spots. While autophagic flux is upregulated, yellow and red spots are both increased. Yellow spots are increased only if the process of autophagosome fusion with lysosomes is inhibited. After treated with apatinib for 24 h, confocal microscopy images showed that the numbers of both yellow dots and red dots increased more apparently in the apatinib group and starvation group than the vehicle group in C643 and KHM-5M cells. We observed that the yellow dots increased and red dots decreased, when the formation of autolysosome formation was inhibited by Baf A1. Yellow dots increased and red dots decreased in apatinib/Baf A1 group than apatinib alone treatment (Fig. 2c, d). Finally, electron microscopy images characteristically showed that ATC cells exposed to apatinib had a higher number of autophagosomes/autolysosomes than the vehicle group (Fig. 2f). Thus, our data strongly suggested that apatinib induced autophagosome formation in ATC cell lines.

### Apatinib induces ATC cell apoptosis

**Fig. 3:**
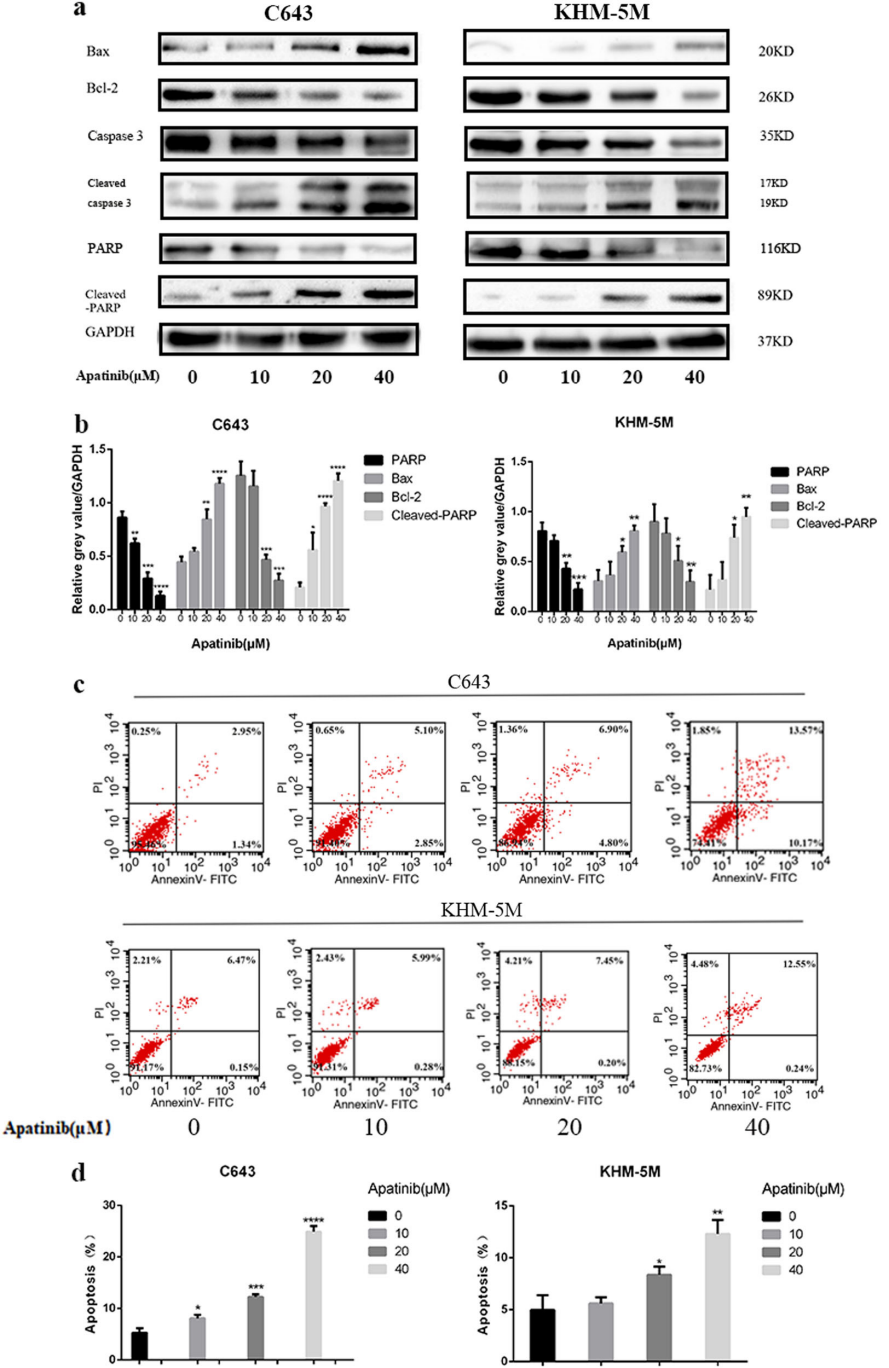
Apatinib promotes apoptosis in ATC cells. **a** C643 and KHM-5M cells were treated with a serious concentration of apatinib for 24 h. The caspase 3, cleaved-caspase 3, PARP, cleaved-PARP, Bcl-2, Bax, and GAPDH expressions were detected by western blot. **b** Quantification of relative gray value of bands compared with GAPDH, as detected by Fig. 3a. **c** C643 and KHM-5M cells were treated with the indicated concentrations for 24 h. The ratio of apoptotic cells was measured by Annexin V-FITC and propidium iodide (PI) staining. The results were representative of three independent experiments. **d** As detected by Fig. 3c, (AnV+) (PI−) cells were considered early apoptotic and (AnV+) (PI+) cells were considered late apoptotic. The columns represent the mean ± SD of the three independent experiments. *p < 0.05, **p < 0.01, ***p < 0.001, ****p < 0.0001 compared with control group

To evaluate the reason behind the growth inhibition induced by apatinib in ATC cells, western blot and flow cytometry were used to detect the level of apoptosis. As evidenced by the western blot analysis, the levels of cleaved-PARP, cleaved-caspase 3 and Bax expression were significantly increased with apatinib for 24 h in ATC cells, whereas Bcl-2, PARP (total), and caspase 3 were consistently decreased (Fig. 3a, b). Flow cytometry was used to analyze the apoptotic cells after Annexin V-FITC and PI staining. Compared with the control group (apatinib 0 µM), treatment with apatinib significantly induced apoptosis in both C643 and KHM-5M cells. After 20 µM apatinib treatment, values that were remarkably higher than those in the control group (C643: 12.23 ± 0.51% vs. 5.26 ± 0.89%, *p* < 0.05 and KHM-5M: 8.36 ± 0.79% vs. 4.98 ± 1.42%, *p* < 0.05) (Fig. 3c, d). In addition, the apoptotic rate increased with respect to the concentrations of apatinib (C643: 24.91 ± 1.1% (apatinib 40 μM), 12.23 ± 0.51% (apatinib 20 μM), 8.08 ± 0.67% (apatinib 10 μM), 5.26 ± 0.89% (apatinib 0 μM); and KHM-5M: 12.3 ± 1.34% (apatinib 40 μM), 8.36 ± 0.79% (apatinib 20 μM), 5.62 ± 0.59% (apatinib 10 μM), 4.98 ± 1.42% (apatinib 0 μM)). Taken together, our results demonstrated that apatinib was able to induce ATC cell apoptosis in a dose-dependent manner.

### The AKT/mTOR signal pathway affects apatinib-induced autophagy and apoptosis

**Fig. 4:**
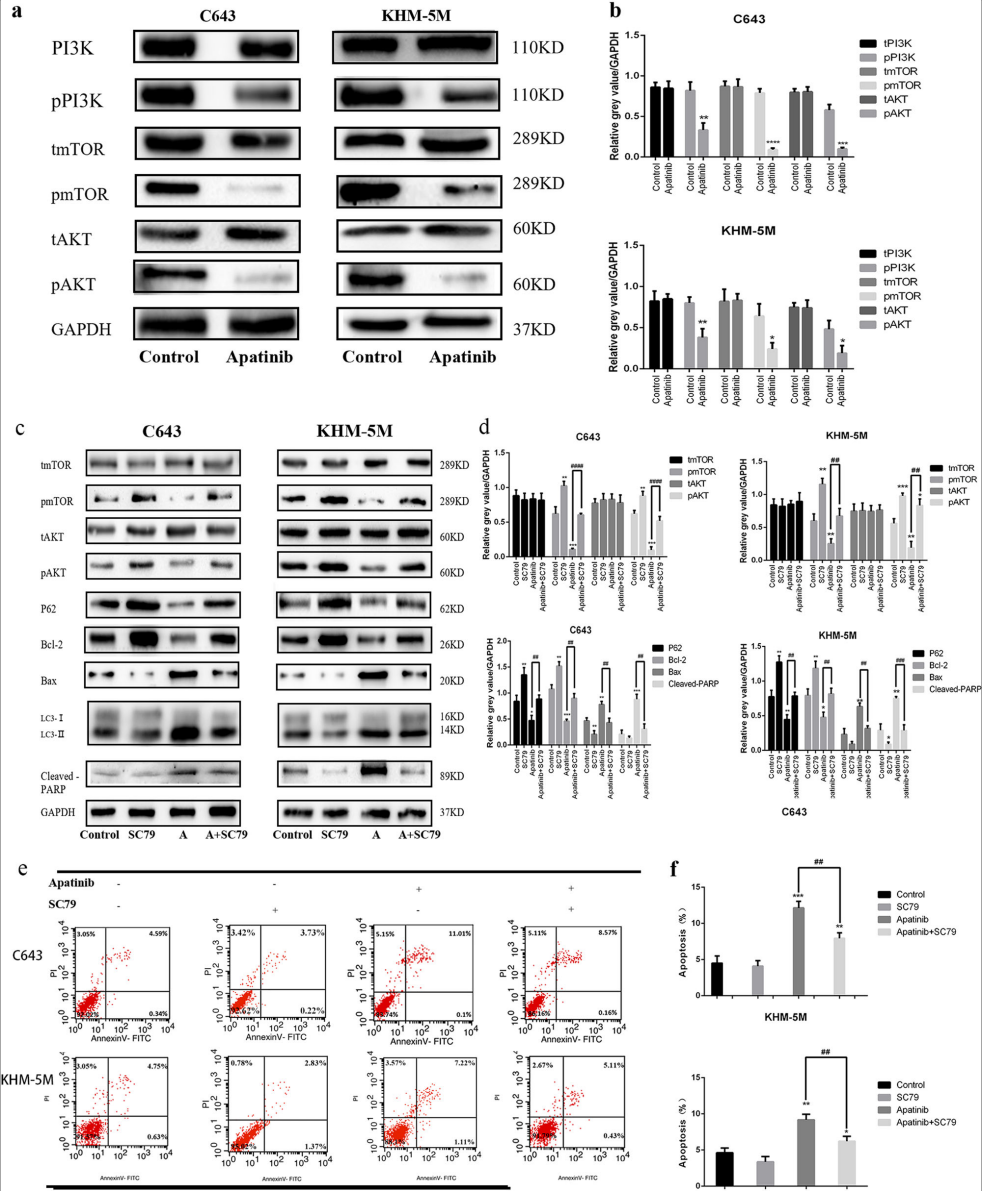

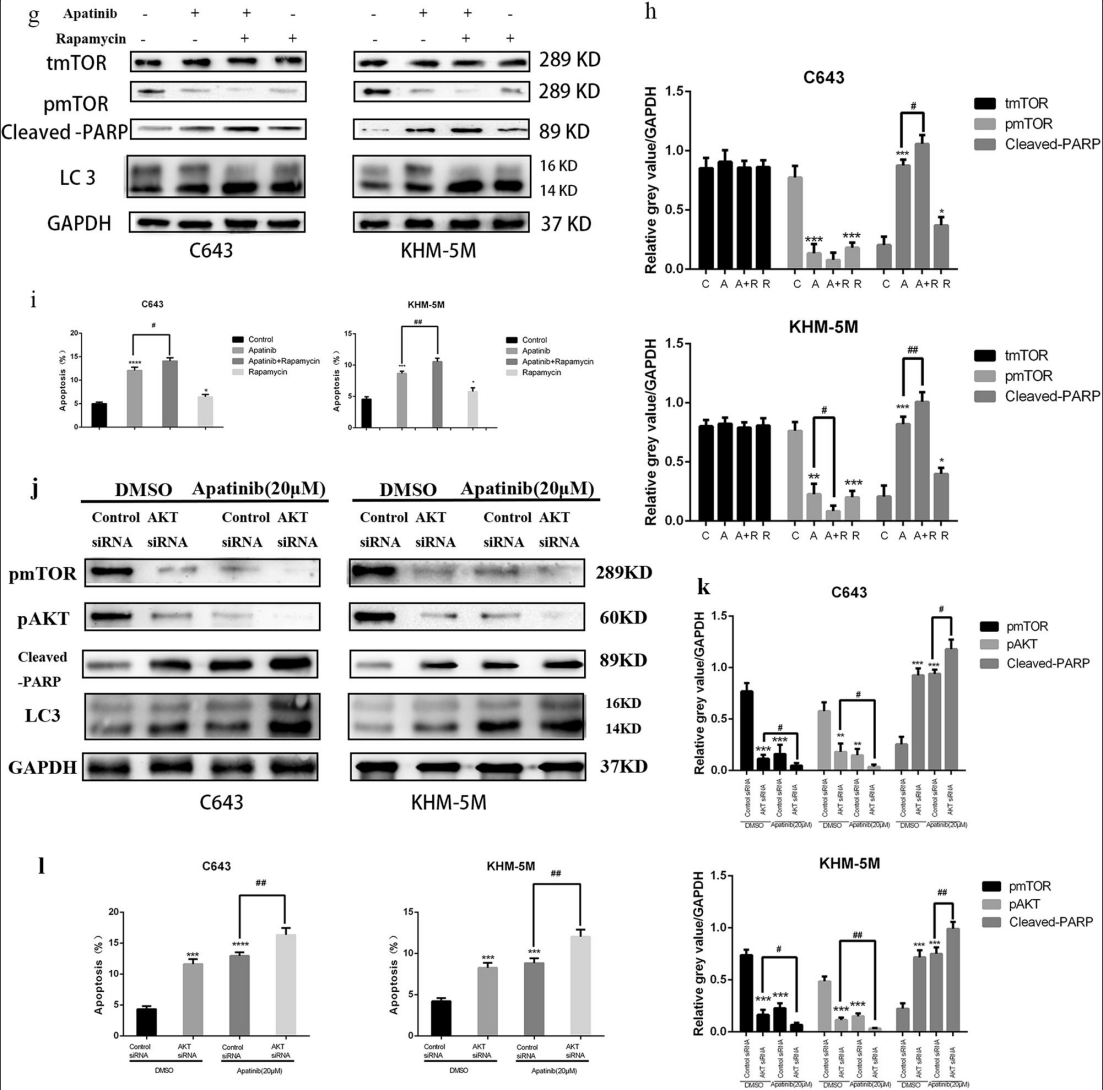
Apatinib induced autophagy and apoptosis via the AKT/mTOR signaling pathway. **a** C643 and KHM-5M cells treated with or without apatinib (20 μM) for 24 h, the expressions of total PI3K, phosphorylated PI3K, total AKT, phosphorylated AKT total mTOR, phosphorylated AKT, and GAPDH detected by western blot. **b** Quantification of relative gray value of bands compared with GAPDH, as detected by Fig. 4a. **c**, **d** C643 and KHM-5M cells were treated by apatinib (20 μM) implement with or without SC79 (4 μg/ml) for 24 h, respectively. Total AKT, phosphorylated AKT, total mTOR, phosphorylated AKT, P62, Bcl-2, Bax, LC3, cleaved-PARP, and GAPDH were detected by western blot. Densitometry represents the expression of the proteins relative to GAPDH. **e**, **f** Cells treated as Fig. 4c, the percentage of apoptotic cells was investigated using Annexin V-FITC and PI. (AnV+) PI− cells were considered early apoptotic and (AnV+) PI+ cells were considered late apoptotic. The columns represent the mean ± SD of the three independent experiments. **g**, **h** Cells were treated by apatinib (20 μM) implement with or without rapamycin (1 μM/l) for 24 h. Western blots of cleaved-PARP, LC3, T-AKT, and p-mTOR extracts from C643 and KHM-5M. **i** After cells were treated as Fig. 4g, the percentage of apoptotic cells was determined using the annexin V and PI staining assay. Data are represented as mean ± SD of three independent experiments. **j** C643 and KHM-5M cells were transfected with control-siRNA and AKT siRNA, respectively, and then were treated with apatinib (20 µM) or DMSO for another 24 h, the expressions of total mTOR, phosphorylated mTOR, cleaved-PARP, LC3, and GAPDH were detected by western blot. **k** Quantification of relative gray value of bands compared with GAPDH, as detected by Fig. 4j. **l** Cells treated as Fig. 4j, the percentage of apoptotic cells was investigated using Annexin V-FITC and PI. The columns represented the mean ± SD of the three independent experiments. *p < 0.05; **p < 0.01, ***p < 0.001, ****p < 0.0001 compared with control. ^#^*p* < 0.05; ^##^*p* < 0.01, ^###^*p* < 0.001, ^####^*p* < 0.0001 (C = control, A = apatinib, R = rapamycin, A + SC79 = apatinib + SC79, A + R = apatinib + rapamycin)

Many reports have suggested that the AKT–mTOR signaling pathway is a major negative regulator of apoptosis and autophagy^28,29^. Western blot results showed that pPI3K, p-AKT and p-mTOR decreased as a result of treatment by 20 µM apatinib for 24 h in both C643 and KHM-5M cells, despite the unchanged total amount of PI3K, AKT and mTOR proteins (Fig. 4a, b). To further detect whether the AKT/mTOR pathway is involved in apatinib-induced autophagy and apoptosis in ATC cells, C643 and KHM-5M were subsequently treated together with apatinib and SC79 (an activator of AKT). As shown in Fig. 4c, co-treatment with apatinib and SC79 significantly reduced Bax, cleaved-PARP, and LC3 expression, whereas p-AKT, p-mTOR, Bcl-2, and P62 expression increased, indicating apoptosis and autophagy process inhibited when compared with apatinib treatment alone (Fig. 4c, d). Flow cytometry data showed that cells treated with apatinib and SC79 have a lower apoptotic rate than cells treated with apatinib alone (C643: 7.95 ± 0.76% vs. 12.14 ± 0.9%, *p* < 0.05; and KHM-5M: 6.21 ± 0.68% vs. 9.18 ± 0.78%, *p* < 0.05, Fig. 4e, f). Hence, activation of AKT via SC79 treatment could restore the autophagy and apoptosis induced by apatinib. Furthermore, rapamycin, an inhibitor of mTOR, was used to verify the involvement of mTOR in the autophagy and apoptosis induced by apatinib. The increasing expression of LC3 lipidation and cleaved-PARP induced by apatinib could be further enhanced by rapamycin treatment (Fig. 4g, h). Flow cytometry shows that ATC cells treated with apatinib and rapamycin had a higher apoptotic rate than apatinib alone (C643: 14.08 ± 0.69% vs. 12.08 ± 0.69%, *p* < 0.05; and KHM-5M: 10.54 ± 0.55% vs. 8.66 ± 0.37%, *p* < 0.05) (Fig. 4i). AKT siRNA was used to knockdown AKT expression in C643 and KHM-5M cells, the expression of LC3 lipidation and cleaved-PARP in control group were lower than AKT siRNA group with or without Apatinib, respectively (Fig. 4j, k). Flow cytometry data showed that apoptotic rates in cells treated with apatinib in AKT siRNA group were greater than those in cells treated with apatinib in control-siRNA group (C643 control-siRNA/apatinib group vs. AKT siRNA/apatinib group 12.94 ± 0.57% vs. 16.33 ± 1.13%, *p* < 0.05; and KHM-5M control-siRNA/apatinib group vs. AKT siRNA/apatinib group 8.82 ± 0.60% vs. 12.04 ± 0.84%, *p* < 0.05) (Fig. 4l). Thus, our results confirmed that the AKT/mTOR signaling pathway could have a vital role in apatinib-induced apoptosis and autophagy regulation.

### Inhibition of autophagy enhances apatinib-induced apoptosis

**Fig. 5:**
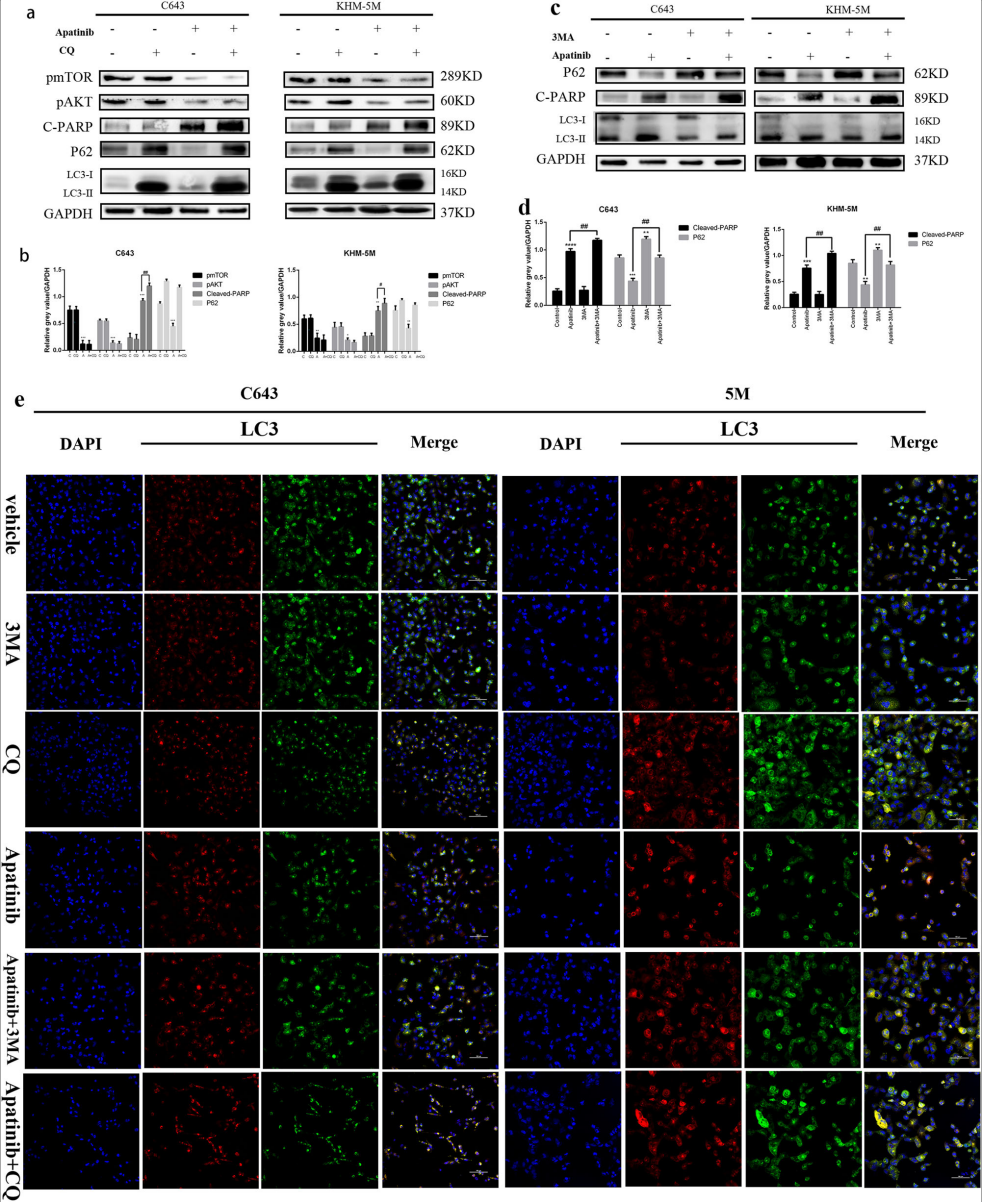

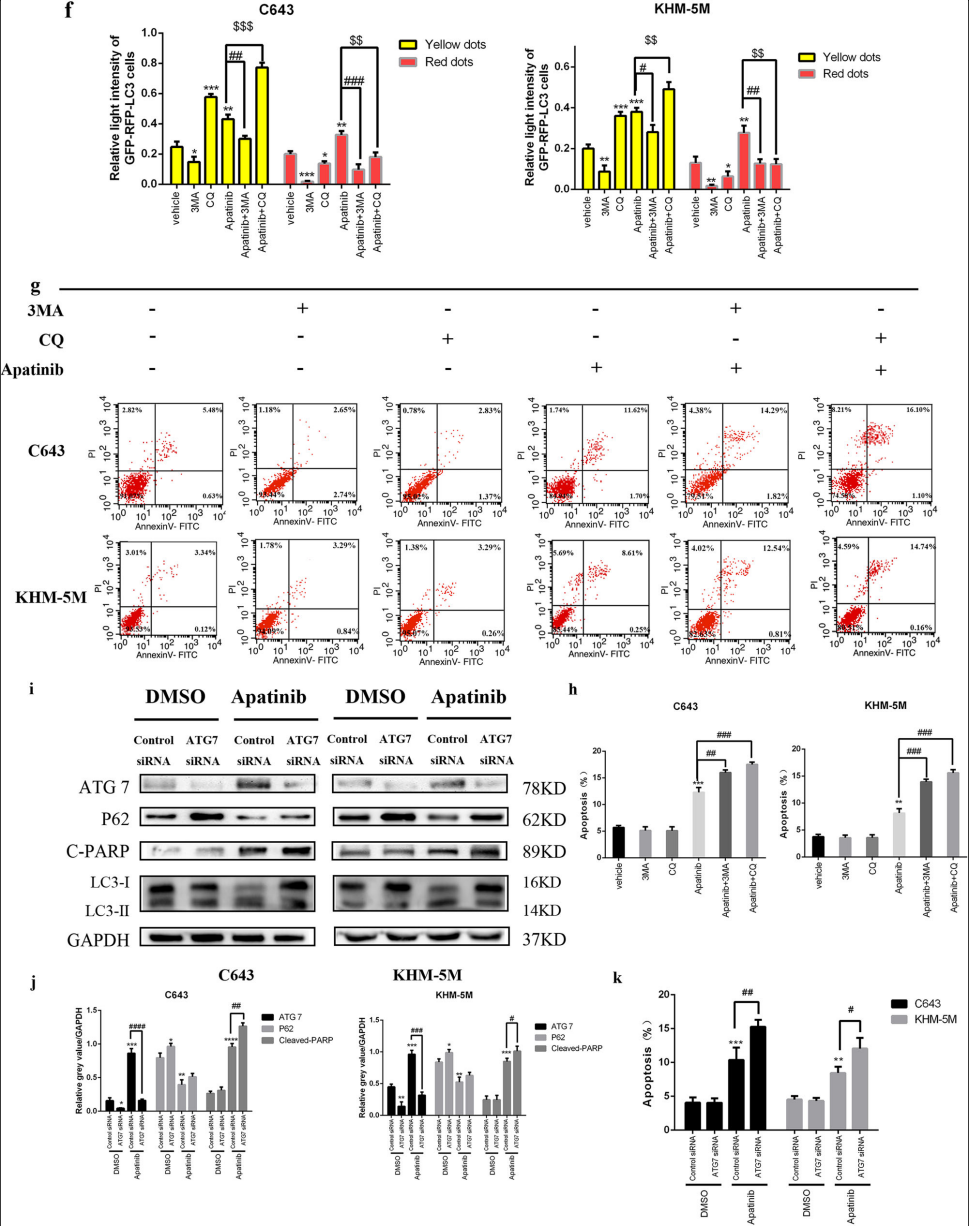
Apatinib-induced autophagy protects ATC cells from apoptosis. **a** C643 and KHM-5M cells were pre-treated with CQ (10 µM) for 6 h, and then with apatinib (20 µM) for another 24 h, p-mTOR, p-AKT, cleaved-PARP, P62, LC3, and GAPDH were detected by western blot. **b** Quantification of relative gray value of bands compared with GAPDH, as detected by Fig. 5a. **c** C643 and KHM-5M cells were treated with apatinib for 24 h in the presence of 3MA(3 mM/l), and then detected cleaved-PARP, LC3, P62, and GAPDH. **d** Quantification of relative gray value of bands compared with GAPDH, as detected by Fig. 5c. **e** As described in Fig. 2c, C643 RFP-GFP-LC3 and KHM-5M RFP-GFP-LC3 cells (transfected with the RFP-GFP-hLC3 lentivirus) were treated with or without apatinib for 24 h in either the presence or absence of 3MA and CQ. **f** Densitometric analysis of the picture in Fig. 5e. **g** Cells treated as Fig. 5e, the percentage of apoptotic cells was investigated using Annexin V-FITC and PI. (AnV+) PI− cells were considered early apoptotic and (AnV+) PI+ cells were considered late apoptotic. The columns represent the mean ± SD of the three independent experiments. **h** Cells treated as Fig. 5e, the percentage of apoptotic cells was determined using the annexin V and PI staining assay. Data are represented as mean ± SD of three independent experiments. **i**, **j** Western blot analyses showed the expression of ATG7, P62, cleaved-PARP, LC3, and GAPDH in C643/ATG7-siRNA and KHM 5M/ATG7-siRNA treated with or without apatinib for 24 h, compared with C643/control-siRNA and KHM 5M/ATG7-siRNA, respectively. **i** Data was shown as the percentage of apoptotic cells. Data were represented as mean ± SD of three independent experiments. *p < 0.05; **p < 0.01, ***p < 0.001, ****p < 0.0001 compared with control. ^#^*p* < 0.05; ^##^*p* < 0.01, ^###^*p* < 0.001, ^####^*p* < 0.0001 (c = control, A = apatinib, A + CQ = apatinib + CQ)

**Fig. 6:**
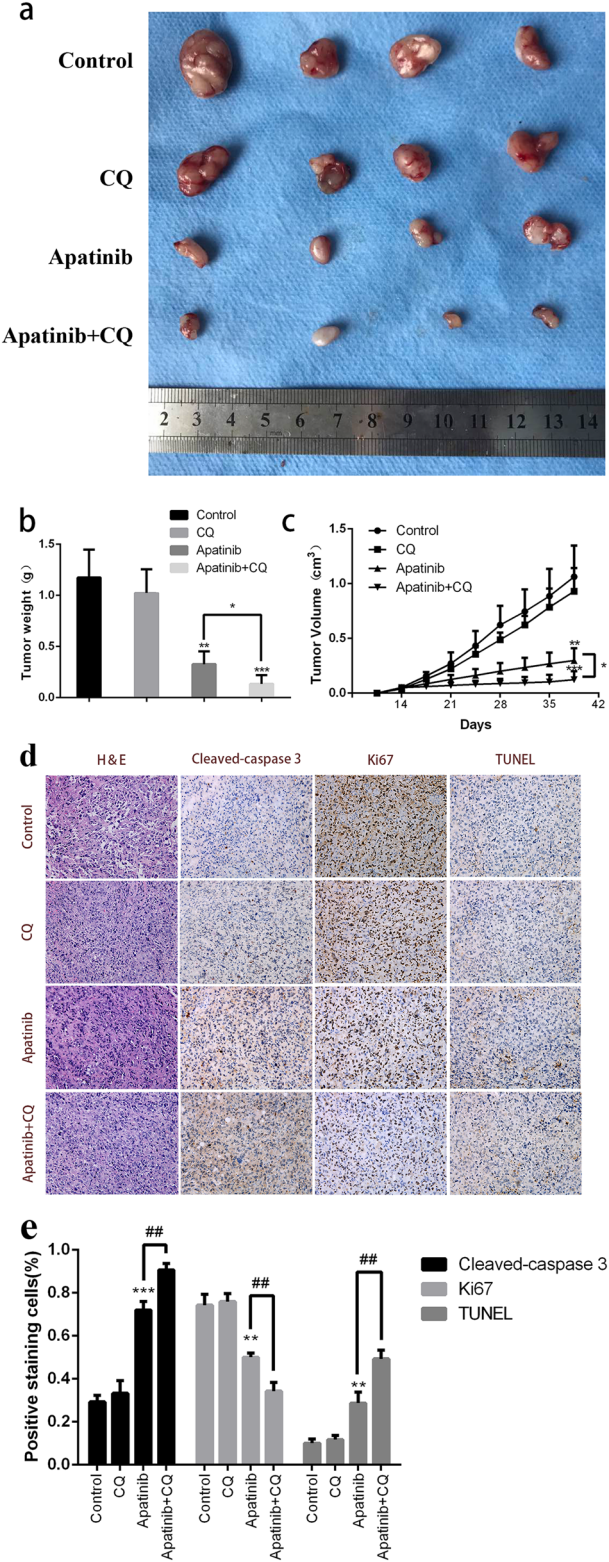
Inhibition of tumor growth in vivo by apatinib and CQ. **a** Typical images of the xenograft tumors. **b** Average tumor weight for each group was calculated. **c** Xenograft tumor volumes were measured twice per week. **d** Representative images of IHC staining of Ki-67 and cleaved-caspase-3 were performed on serial sections of tumors from KHM-5M/Control, KHM-5M/CQ, KHM-5M/apatinib, and KHM-5M/apatinib plus CQ group, and cell apoptosis was assessed by TUNEL assay. **e** Densitometric analysis of the picture in Fig. 6d. *p < 0.05; **p < 0.01, ***p < 0.001, ****p < 0.0001 compared with control. ^#^*p* < 0.05; ^##^*p* < 0.01, ^###^*p* < 0.001, ^####^*p* < 0.0001

The paradoxical function of autophagy in the tumor might cause autophagic tumor cell death or adaptation to drug cytotoxicity, and hence, autophagy can support or inhibit the proliferation of tumor cells in different scenarios^18,19^. CQ, an antimalarial lysosomal inhibitor, has been identified as an inhibitor of autophagy, which can prevent effective autophagic degradation and lead to accumulation of ineffective autophagosomes^30,31^. Both LC3 lipidation and P62 could be upregulated as a result of autophagosome accumulation due to the inhibition of the autophagy effective stage. As shown in Fig. 5a and b, the western blot results showed that CQ pre-treatment could increase LC3 lipidation and p62 as expected, indicating autophagy inhibition. Additionally, compared with apatinib solo treatment, apatinib combined with CQ pre-treatment led to an increase of cleaved-PARP expression, revealing increased apoptosis in addition to autophagy inhibition. 3MA(3-Methyladenine) was also used to inhibit autophagy induced by apatinib in C643 and KHM-5M cell lines. Figure 6c and d showed the expression of p62 and cleaved-PARP were increased and LC3 lipidation was decreased with 3MA-apatinib treatment than apatinib solo. The results above illustrated that inhibition of autophagy induced by apatinib could enhanced apoptosis in ATC cells. Confocal microscopy images in Fig. 5e and f demonstrated fewer red spots and more yellow spots (autophagosome fusion with lysosomes is inhibited) in the apatinib with CQ pre-treatment group, and fewer yellow and red dots were observed in 3MA-apatinib group than apatinib group. Data from flow cytometry proved that apatinib plus pre-treatment with CQ induced greater apoptosis rates (apatinib/CQ vs. apatinib: C643: 17.51 ± 0.44% vs. 12.28 ± 0.90%, *p* < 0.05; KHM-5M: 15.57 ± 0.61% vs. 8.13 ± 0.83%, *p* < 0.05). Similarly, 3MA-apatinib treatment induced more apoptosis rate than apatinib single (apatinib/3MA vs. apatinib: C643: 15.98 ± 0.51% vs. 12.28 ± 0.90%, *p* < 0.05; KHM-5M: 13.92 ± 0.50% vs. 8.13 ± 0.83%, *p* < 0.05) (Fig. 5g, h). Altogether, our results indicated that apatinib, by means of CQ and 3MA treatment, could indeed enhance apoptosis together with autophagy inhibition. Furthermore, ATG7-siRNA was used to inhibit the early stage of autophagosome formation. The western blot results in Fig. 5i confirmed that the expression of ATG7 was diminished effectively in both KHM-5M and C643 cells. In addition to the p62 increase, the LC3 lipidation decrease demonstrated the special inhibition of autophagy induced by apatinib. Furthermore, following autophagy inhibition, cleaved-PARP expression was increased. Comparing apatinib with the control-siRNA group, flow cytometry proved that apatinib with ATG7-siRNA induced greater apoptosis rates (apatinib/ATG7-siRNA vs. apatinib: C643: 15.27 ± 1.04% vs. 11.37 ± 0.84%, *p* < 0.05; KHM-5M: 12.06 ± 1.57% vs. 8.44 ± 0.92%, *p* < 0.05), while without apatinib treatment, the apoptosis rates of both group had no obvious difference (ATG7-siRNA vs. control-siRNA: C643: 4.06 ± 0.79% vs. 4.05 ± 0.65%, *p* > 0.05; KHM-5M: 4.53 ± 0.51% vs. 4.32 ± 0.46%, *p* > 0.05) (Fig. 5i–k). These results revealed that transfection with ATG7-siRNA could significantly enhance the apoptosis induced by apatinib in both C643 and KHM-5M cells. Thus, our results indicated that inhibiting the early stage of autophagosome formation not only could prohibit autophagy launch but could also enhance apoptosis in ATC cells. In all, although 3MA, CQ, and ATG7 could block autophagy activation in different steps, apoptosis could be promoted, which might be related to the deceleration of ATC cell proliferation.

### Tumor suppression induced by apatinib is enhanced by CQ

Based on the results above, xenograft models were used to further confirm the effect of apatinib-mediated autophagy and apoptosis on tumor proliferation in vivo. After KHM-5M cells were subcutaneously injected into the right flanks, nude mice were divided into four groups: group 1 = vehicle control, group 2 = CQ solo treatment, group 3 = apatinib solo treatment, and group 4 = apatinib with CQ treatment. The xenograft tumors were allowed to develop for 14 days after injection, and the corresponding treatment was started according to therapeutic comparison. At 26 days after treatment, all mice were sacrificed. As expected, the tumor volumes in the CQ treatment did not show a significant difference (CQ vs. vehicle: 93 ± 21.2 mm^3^ vs. 106.25 ± 28.45 mm^3^, *p* > 0.05) compared with those in the vehicle control group. The CQ combined with apatinib group exerted greater antitumor effects in KHM-5M xenograft tumor models compared with the drugs administered independently. (apatinib + CQ vs. apatinib: 12.25 ± 7.68 mm^3^ vs. 29.75 ± 11.32 mm^3^, *p* < 0.05; apatinib + CQ vs. CQ: 12.25 ± 7.68 mm^3^ vs. 93 ± 21.2 mm^3^ *p* < 0.001) (Fig. 6a–c). Consistent with the results of tumor volume, tumor weight could be suppressed by apatinib mono-therapy or CQ + apatinib combination therapy. Apatinib combined with CQ treatment showed a more effective inhibition (apatinib + CQ vs. apatinib: 13.48 ± 8.44 mg vs. 32.73 ± 12.46 mg, *p* < 0.05), and the percentage of growth inhibition in CQ group, apatinib group and CQ/apatinib group were 12.9%, 72.1%, and 88.5%, respectively (Fig. 6b). Moreover, the expressions of Ki-67, and cleaved-caspase 3 in xenografts were detected by immunohistochemistry, and cell apoptosis was assayed by TUNEL. Compared with any other group, the expressions of Ki-67 was significantly reduced in the apatinib/CQ group, and the levels of cleaved-caspase 3 were significantly increased in the apatinib/CQ group. These results were further confirmed by the TUNEL assay (Fig. 6d, e). Indeed, the apatinib/CQ group had the greatest effect of tumor suppression among the four groups. All results showed that inhibited autophagy could enhance the effect of apatinib-induced growth inhibition in ATC cells in vivo.

## Discussion

Although ATC has a rather low incidence, it was always characterized with a high risk of recurrence and poor prognosis. Traditional surgical treatment has been proven ineffective for ATC. Standard chemotherapy and targeted therapy were considered as the only palliative treatment method, but they did not prolong the 5-yr survival rate significantly^2–4,32^. Apatinib, a highly selective tyrosine kinase inhibitor of VEGFR-2, was proven to have an anti-tumor effect in various tumors^5–7,33^. Recently, apatinib has demonstrated a promising therapeutic effect for radioiodine refractory differentiated thyroid cancer^34^, which suggests a novel method for ATC treatment. We previously confirmed that apatinib could significantly reduce ATC angiogenesis in a dose- and time-dependent manner. Additionally, apatinib could decrease the ATC proliferation ability both in vivo and in vitro^23^. In addition to angiogenesis inhibition, in this study, apatinib could activate apoptosis and autophagy via suppression of the AKT/mTOR pathway, and apatinib-induced apoptosis could be enhanced by inhibiting apatinib-induced autophagy. This novel regulation mechanism could underline the anti-tumor effect of apatinib in ATC treatment.

Recent studies suggest that the autophagy and apoptosis signaling pathways can interact with each other^34–38^. Inhibition of autophagy could enhance apoptosis in osteosarcoma cells^39^, and autophagy promotes apoptosis in mesenchymal stem cells under inflammatory microenvironment^40^. Therefore, understanding interaction of apoptosis and autophagy may be conducive to launch a new therapeutic strategy for ATC^41^. In our present study, we demonstrated that both autophagy and apoptosis in ATC cells could be enhanced by apatinib treatment. 3MA, CQ and ATG7-siRNA, chemical and genetic autophagy inhibitors, could, respectively, restrain autophagy in late phase and early phase; hence, they were employed to portrait the interactive relationship between autophagy and apoptosis. The results showed apatinib-induced autophagy could be effectively abolished by 3MA, CQ, and ATG7-siRNA. Consequently, blocking autophagy could promote apoptosis in ATC cell lines, which indicated that anti-proliferative effect of apatinib could be significantly boosted on autophagy inhibition. Thus, our study revealed the vital role of autophagy and apoptosis interaction in ATC therapeutic resistance.

The AKT signaling pathway is one of the major survival gateways of tumor cells, and growing evidence has showed that the over-active AKT/mTOR pathway promotes tumor cell survival^42–45^. As a downstream of VEGFR, PI3K–AKT–mTOR signaling pathway is deeply involved in apoptotic effect of VEGFR-targeting therapy^46,47^. A promising therapeutic effect of apatinib in our current study has indicated the efficacy of VEGFR-2 inhibition in ATC (Supplementary material). Apatinib could decrease p-AKT and p-mTOR level in ATC cells. SC79, a special agonist for AKT, could restore the autophagy and apoptosis induced by apatinib in ATC. Similarly, rapamycin was used to reduce p-mTOR expression. Furthermore, AKT siRNA was used to knockdown the expression of AKT and p-AKT. Results above shown that apatinib could regulate the AKT/mTOR signaling pathway in ATC. Furthermore, inhibition of AKT–mTOR signaling pathway could directly stimulate autophagy and apoptosis^48,49^. In our study, apatinib-induced autophagy and apoptosis were decreased by SC79, and through downregulation the expression of p-AKT by AKT siRNA, the autophagy and apoptosis induced by apatinib were enhanced. mTOR is mainly associated with apoptosis and autophagy. Rapamycin co-treatment has a modest increase in autophagy and apoptosis, but inhibiting autophagy with apatinib treatment will cause a greater increase. Thus, we confirmed that apatinib induce autophagy and apoptosis via AKT/mTOR signaling pathway, and AKT/mTOR signaling pathway may closely associate with the growth inhibition effects of apatinib in ATC. Moreover, autophagy inhibitors as potential adjuvants to apatinib might represent an attractive therapeutic strategy for ATC treatment.

In conclusion, our data revealed a novel mechanism whereby apatinib induced apoptosis and autophagy via AKT/mTOR pathway in ATC cell lines. Meanwhile blocking autophagy could enhance anti-tumor effect of apatinib in ATC cell lines both in vitro and in vivo. These findings validate that targeting autophagy with apatinib could be a promising therapeutic strategy for ATC.

## Electronic supplementary material

Supplementary figure
