# Supplementary material for: Apatinib-induced protective autophagy and apoptosis through the AKT–mTOR pathway in anaplastic thyroid cancer
Source: Cell Death Dis. 2018 Oct 9;9(10):1030. doi: 10.1038/s41419-018-1054-3 (PMC6177436; doi:10.1038/s41419-018-1054-3)

**Apatinib-induced protective autophagy and apoptosis through the AKT-mTOR pathway in anaplastic thyroid cancer**

Haoran Feng*^1,2^, Xi Cheng*^1,2^, Jie Kuang*^1,2^, Lingxie Chen^1,2^, Stanley Yuen^3^, Minmin Shi^1,2^, Juyong Liang^1,2^, Baiyong Shen^1,2^, Zhijian Jin**^1,2^, Jiqi Yan**^1,2^, Weihua Qiu**^1,2^.

1. Department of General Surgery, Ruijin Hospital, Shanghai Jiao Tong University School of Medicine, Shanghai, China, 200025

2. Shanghai Institute of Digestive Surgery, Ruijin Hospital, Shanghai Jiao Tong University School of Medicine, Shanghai, China, 200025

3. Biology Chemistry Major, University at Albany, New York, NY, USA

Financial support: Natural Science Foundation of China (NSFC: 81772558), Shanghai Charity Research Foundation of Cancer Research, Ph.D. Innovation Fund of Shanghai Jiaotong University School of Medicine (BXJ201709) and “Visiting Programs for Graduate Students of Shanghai Jiaotong University School of Medicine”.

* These authors have contributed equally to this study

**Supplementary information**

**Supplementary Figure legend**

**Supplementary Figure** **1**:Western blot analyses showed the expression of pVEGFR2, HIF-1α, P65, pP65, P53 and pP53 in C643 and KHM5M treated with different concentration of apatinib for 24 h (0, 20, 40μM)


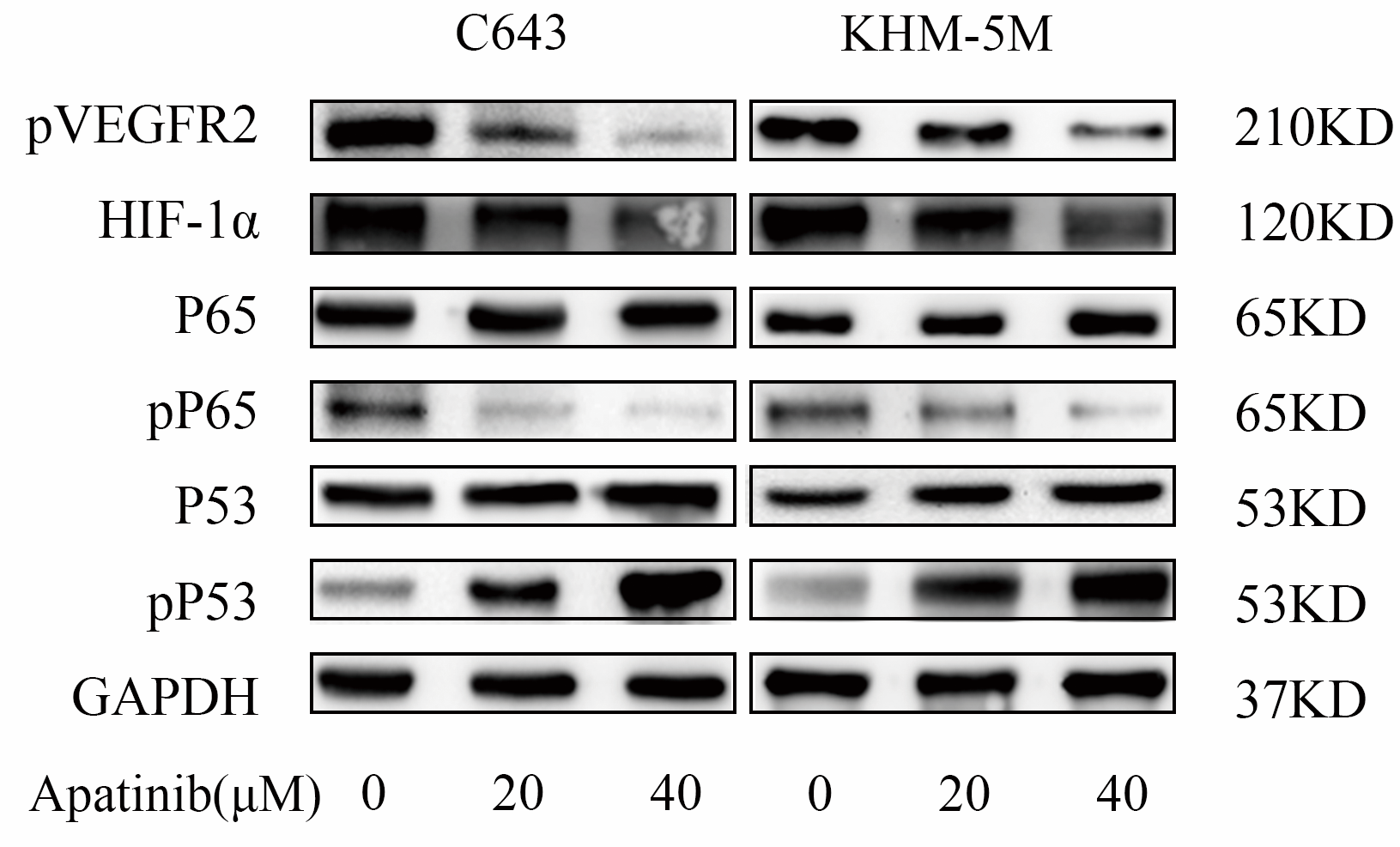

Supplement: Supplementary file 1 — Supplementary figure [file 41419_2018_1054_MOESM1_ESM.docx]
